# Research Progress in Skin Aging, Metabolism, and Related Products

**DOI:** 10.3390/ijms242115930

**Published:** 2023-11-03

**Authors:** Xin He, Xinyu Gao, Weidong Xie

**Affiliations:** 1 State Key Laboratory of Chemical Oncogenomics, Shenzhen International Graduate School, Tsinghua University, Shenzhen 518055, China; hexin22@mails.tsinghua.edu.cn (X.H.); gao-xy23@mails.tsinghua.edu.cn (X.G.); 2 Open FIESTA Center, Shenzhen International Graduate School, Tsinghua University, Shenzhen 518055, China; 3 Shenzhen Key Laboratory of Health Science and Technology, Institute of Biopharmaceutical and Health, Tsinghua University, Shenzhen 518055, China

**Keywords:** skin aging, glucose metabolism, protein metabolism, lipid metabolism, anti-skin-aging drugs

## Abstract

In recent years, skin aging has received increasing attention. Many factors affect skin aging, and research has shown that metabolism plays a vital role in skin aging, but there needs to be a more systematic review. This article reviews the interaction between skin metabolism and aging from the perspectives of glucose, protein, and lipid metabolism and explores relevant strategies for skin metabolism regulation. We found that skin aging affects the metabolism of three major substances, which are glucose, protein, and lipids, and the metabolism of the three major substances in the skin also affects the process of skin aging. Some drugs or compounds can regulate the metabolic disorders mentioned above to exert anti-aging effects. Currently, there are a variety of products, but most of them focus on improving skin collagen levels. Skin aging is closely related to metabolism, and they interact with each other. Regulating specific metabolic disorders in the skin is an important anti-aging strategy. Research and development have focused on improving collagen levels, while the regulation of other skin glycosylation and lipid disorders including key membrane or cytoskeleton proteins is relatively rare. Further research and development are expected.

## 1. Introduction

The skin is considered the most extensive organ system in mammals [1]; making up 10% of body weight, the skin needs to rely on material and energy metabolism to maintain structural and functional integrity. In recent years, with the improvement in people’s living standards and the increase in the aging population, people, especially women, have begun to pay attention to the topics of skin aging and anti-aging [2,3]. Changes in the skin barrier occur during skin aging [4]. Aging skin exhibits wrinkles, uneven skin tone [5,6], a loss of elasticity, and thinning [7]. There are many factors that affect skin aging. Generally speaking, skin aging problems naturally occur with age, and skin aging is also influenced by both internal (genetics and diseases) and external factors (ultraviolet radiation, air pollution, smoking, alcohol consumption, etc.), leading to accelerated aging [8,9,10].

The skin’s structural complexity involves a diverse assembly of specialized cell types, including keratinocytes, fibroblasts, melanocytes, and immune cells, which are all strategically organized to execute their unique roles within the skin’s intricate framework [11]. For instance, keratinocytes, the predominant cell type in the epidermis, form a protective barrier against environmental factors and pathogens [12]. Fibroblasts, residing in the dermal layer, play crucial roles in synthesizing extracellular matrix components, particularly collagen and elastin, which are responsible for skin elasticity and firmness [13]. Melanocytes are responsible for skin pigmentation, and immune cells act as sentinels, defending the skin against infections and injuries [14].

Unveiling the intricate process of aging and its impact on the skin demands a multifaceted approach. Beyond the external factors shaping skin aging, a profound understanding of the skin’s internal architecture is pivotal. Currently, various types of skin cells, such as keratinocytes, fibroblasts, and melanocytes, are well identified and, as described above, play significant roles in the structure and function of the skin [15]. With the passage of time, changes in the cellular and structural elements in the skin manifest as aging characters, e.g., wrinkles, reduced elasticity, and pigmentation fluctuations. Comprehending the complexities of skin aging hinges on a grasp of the skin’s biological procession. Intertwined within this narrative lies the symbiotic relationship between skin biology and metabolism, a key element in unraveling the mystery of skin aging. The interplay of the skin’s biology, which is intricately woven with metabolic processes, constitutes the foundational bedrock upon which we embark our quest to comprehend and combat the effects of aging on our skin.

Metabolism, in general, refers to a series of organized chemical reactions that occur within an organism to sustain life. These metabolic processes enable organisms to grow, reproduce, maintain their structures, and respond to the external environment. Metabolism is categorized into material metabolism and energy metabolism, with material metabolism primarily encompassing three major metabolites: glucose metabolism, protein metabolism, and lipid metabolism. Metabolism plays a vital role in maintaining physiological functions and influences the occurrence and development of diseases within the body [16]. Furthermore, it is essential to establish a clear connection between metabolism and specific cell types within the skin. This linkage not only fortifies the overarching narrative of our study but also furnishes crucial context for our subsequent discussions.

Recent studies have revealed a close connection between skin aging and metabolism, with both factors exerting significant reciprocal influences. For example, glucose, protein, and lipid metabolism can all impact the skin’s aging process. Glucose and galactose in glucose metabolism are directly linked to skin aging, and issues with glucose metabolism can hasten the aging of the skin [17]. The aging of the skin can contribute to a deceleration in glucose metabolism, resulting in the accumulation of skin glycation and further worsening the aging process. The most abundant proteins in the skin include keratin, collagen, and elastin. Reduced collagen synthesis and increased metabolism can promote skin aging. In addition, the secretion of oil that protects the skin is no longer strong, and the low lipid content of the skin can also lead to skin aging [18,19,20].

Given the above, we can observe a significant correlation between skin aging and metabolism. Studying the relationship between skin metabolism and aging, as well as exploring anti-aging strategies based on skin metabolic disorder regulation, has practical research significance. Next, this article will review the interaction between skin metabolism and aging, as well as anti-aging strategies and effective drugs based on metabolic regulation, in the hope of providing new references for the discovery of anti-skin-aging strategies and related drugs.

## 2. The Impact of Skin Aging on Metabolism

### 2.1. Skin Metabolism

Skin metabolism mainly includes the metabolism of three major substances: glucose, protein, and lipid metabolism. During skin aging, the related metabolism will be disrupted or altered.

#### 2.1.1. Glucose Metabolism

Glucose metabolism refers to a series of complex chemical reactions that occur in the body between glucose and glycogen. The main forms of glucose that are present in the body are glucose and glycogen. Glucose metabolism includes catabolism and anabolism [21]. Glycolysis metabolism refers to the breakdown of glucose into small substances, which undergo a series of decomposition in the organism, releasing a large amount of energy and forming large carbon shelves such as lipids, proteins, and nucleic acids. Glycosynthesis metabolism is the process by which organisms convert certain non-glucose small molecules into monosaccharides and polysaccharides, which requires energy.

Glucose is the main fuel for skin energy production. A total of 70% of glucose in the human epidermis is converted into lactic acid through anaerobic glycolysis [22]. Lactic acid can be directly secreted into the body to exert antibacterial effects, while also entering the bloodstream to participate in gluconeogenesis. Only 2% of glucose participates in complete aerobic glucose metabolism through the tricarboxylic acid cycle. The pentose phosphate metabolism pathway of glucose is common in the skin and plays an important role in the growth and repair of skin tissue cells. Glycogen is synthesized from glucose monomers and plays an energy storage role in the skin. When glucose is lacking in energy, the distribution of fuel is redistributed, and the skin’s energy source shifts to other fuels such as glycogen or lipids [23].

In aging skin, glucose metabolism is disrupted. Research has shown that the metabolism of pentose phosphate in aging skin cells decreases. Glucose metabolism may be slowed down, resulting in a decrease in the overall metabolic efficiency, a high local glucose concentration in the skin, and susceptibility to glycation, leading to increased AGE production and damage to the skin and accelerating the process of skin aging [24]. At the same time, the disorder of skin glucose metabolism can affect the breakdown of skin proteins and lipids, affect the structure and function of the skin, and lead to aging.

#### 2.1.2. Protein Metabolism

Protein metabolism refers to the biochemical processes of proteins and amino acids, including synthetic metabolism and catabolism. During the process of protein catabolism, amino acids can be produced through protease degradation [25].

The metabolism of skin proteins is an indispensable and important process for maintaining skin function [26], and the integrity of skin tissue largely depends on its protein matrix. The primary function of collagen is to maintain skin homeostasis, and it can appropriately regulate the structure of the skin matrix, which is a crucial role in skin homeostasis [27]. In aging skin, the most obvious changes in protein metabolism are a decrease in collagen synthesis and an increase in collagen degradation, which lead to a decrease in the amount of collagen [28]. Type I collagen is the most abundant type of collagen and the main structural protein in human tissues [29,30]. Aging can lead to a decrease in the synthesis of type I collagen and elastin in fibroblasts.

Glutamine has been proven to be the amino acid with the highest content in plasma and muscle [23]. Glutamine can serve as a donor for energy and nitrogen sources required for purine/pyrimidine formation, participating in mitochondrial metabolism and cell growth regulation [12]. During hunger stress, glucose utilization is hindered, and protein is degraded into glutamine or becomes an important energy source for skin tissue [23]. During wound healing, epidermal cells divide and grow, and glutamine is an important growth factor. Glutamine can be degraded by glutaminase to produce glutamate, which can be converted into α- Ketoglutaric acid participates in the tricarboxylic acid cycle as a replenishment substrate [21]. Therefore, glutaminase is related to skin aging. Studies have used mouse skin and discovered that glutaminase activity decreases with age, resulting in a slower metabolism of glutamine, which may affect the structure and function of the skin [22].

#### 2.1.3. Lipid Metabolism

Lipids are important components of all cell types and play various biological functions, including energy storage, cell membrane construction, cell transduction, protection, and mitochondrial regulation [31,32]. Fatty acids can be considered as energy fuels in situations where energy is required. The oxidation of fatty acids has been shown to produce acetyl CoA and NADH. Fatty acids not only have important significance in lipid metabolism but also play a very important role in protecting the skin’s epidermal structure [22].

Lipids such as fatty acids are important energy sources for skin tissue cells after glucose. When hungry, skin tissue can utilize fatty acids as a source of energy. In addition, lipids play important roles in supporting the basic structure of skin cells and preventing skin water loss [33,34]. An important characteristic of lipid metabolism in aging skin is a decrease in protective lipid synthesis and secretion. In aging skin, there is a notable slowdown in lipid synthesis and metabolism, resulting in reduced lipids that serve as protective barriers for the skin. This phenomenon contributes to skin thinning and heightens the propensity for an accelerated aging process [35]. Another characteristic of skin aging is the qualitative changes in lipids, such as the susceptibility to peroxidation, which leads to the generation of lipid peroxidation products and the occurrence of aging characters.

## 3. The Impact of Metabolism on Skin Aging

Conversely, the metabolic processes of glucose, proteins, and lipids play crucial roles in skin aging.

### 3.1. The Effect of Glucose Metabolism on Skin Aging

In skin cells, glucose metabolism disorders can have significant impacts on skin aging. On the one hand, the excessive metabolism of glucose in the mitochondria can produce excessive reactive oxygen species (ROS), which can stimulate the body’s stress system and cause skin damage. On the other hand, when glucose metabolism slows down, due to glucose accumulation, proteins and glucose form a cross-link in the body through non-enzymatic glycosylation reactions, producing advanced glycation end products (AGEs) [36], damaging the skin [17], and accelerating skin aging [37]. Currently, AGEs are increasingly being recognized as important factors in studying the issue of skin aging [38], which makes collagen fibers unable to recover after production. Glucose and fructose play pivotal roles as catalysts in triggering this cross-linking effect [39]. The saccharification process is considered one of the key parameters for accelerating the signs of skin aging [40].

### 3.2. The Impact of Protein Metabolism on Skin Aging

Protein serves as a noticeable marker in both healthy skin and aging skin, with a particular emphasis on collagen. Collagen assumes a pivotal role in the examination of skin structural support and the aging process, making it a direct indicator of skin aging. A decline in collagen levels can significantly impact skin quality, contributing to the manifestation of age-related skin characteristics [41].

Glutamine is very instrumental in cell growth, detoxification, and barrier building in the skin [12]. Studies have shown that the homeostasis of glutamine in skin cells plays an important role in metabolism, and the antioxidant capacity and defense against external stimuli in skin cells largely come from glutathione (GSH), which is derived from glutamine [42]. To some extent, the more glutamine available, the easier it is for the skin to remain youthful [43].

### 3.3. The Effect of Lipid Metabolism on Skin Aging

The lipid profile of the skin is the foundation for maintaining a protective barrier to the external environment [44]. A decrease in skin lipid synthesis can lead to the loss of skin moisture and promote the occurrence of skin aging. Ultraviolet radiation may affect the skin’s lipid homeostasis [45], causing lipid peroxidation and the formation of brown pigments, leading to the occurrence and development of skin aging. The metabolic product of lipids, fatty acids, can affect matrix metalloproteinases (MMPs), which affect the amount of collagen produced and directly affect the degree and state of skin aging. Therefore, skin lipid metabolism is also very complex, and its metabolic disorders can also play very important roles in skin aging.

## 4. Metabolism and Aging in Specific Skin Cells

Skin tissue consists of different cells. Specific types of cells are crucial in the field of skin metabolism and aging. These cells include keratinocytes, fibroblasts, and melanocytes, each of which play an indispensable and unique role [8]. Next, we will introduce the metabolic and aging content of three main types of cells in the skin one by one.

### 4.1. Keratinocytes

Keratinocytes constitute the primary component of the skin’s outermost layer—which is crucial for skin protection—and lipid metabolism, which is essential for maintaining the skin’s barrier function. This barrier acts as a protective shield, preventing moisture loss and the infiltration of external substances. Furthermore, protein metabolism within keratinocytes involves the synthesis of keratin, which forms the structural foundation of the epidermal layer. This structural support is instrumental in preventing moisture loss and the intrusion of external elements, thereby bolstering skin health and appearance [46].

Several mechanisms come into play in the context of glucose metabolism within aging keratinocytes. With age, these cells may experience alterations in glucose uptake and utilization. Reduced glucose uptake can impact energy production and oxidative stress management [47]. The cells might also face changes in the expression of critical enzymes that are involved in glycolysis and the citric acid cycle, potentially leading to diminished energy production [48]. Furthermore, aging keratinocytes may exhibit increased levels of oxidative stress, which can disrupt glucose metabolism and hinder the cells’ abilities to counter the damaging effects of environmental factors and free radicals [49].

These metabolic alterations can contribute to the compromised metabolic capacity of keratinocytes during the aging process, resulting in the degeneration of the epidermal layer and the acceleration of skin aging. This increases water loss, rendering the skin more susceptible [50]. In addition to these roles, keratinocytes require ample energy to combat oxidative stress, given the skin’s continuous exposure to environmental factors and free radicals. Maintaining normal glucose metabolism is critical for providing antioxidant protection and preserving overall skin health [47].

### 4.2. Fibroblasts

Fibroblasts, which are situated deep within the skin’s dermal layer, assume a pivotal role in skin health by actively synthesizing essential proteins, mainly collagen and elastin [51]. These structural proteins provide the skin with the necessary framework and elasticity. In youthful skin, fibroblasts exhibit robust metabolic activity, characterized by the prolific synthesis of collagen and a high degree of cellular regeneration [21]. This dynamic activity maintains the skin’s structural integrity and suppleness, contributing to its youthful vitality. However, age brings about changes in the metabolic functions of fibroblasts. As age increases, the metabolic activity of these fibroblasts can wane, ultimately tilting the balance towards increased collagen degradation, surpassing the rate of collagen synthesis [52]. This metabolic shift results in the gradual loss of skin elasticity and the emergence of fine lines and wrinkles.

Fibroblasts are integral to sugar metabolism within the skin. Their ability to maintain stable sugar metabolism is essential for preventing the formation of advanced glycation end products (AGEs) [36]. AGEs are compounds that are formed when proteins, including collagen and elastin, become glycated due to the exposure to excess sugar. The accumulation of AGEs can lead to the cross-linking of proteins and the loss of skin elasticity, contributing to visible signs of aging such as wrinkles and sagging [53]. Therefore, the age-related changes in the metabolic activities of fibroblasts can have direct impacts on the formation of AGEs and subsequently affect the skin’s aging process.

Furthermore, fibroblasts are at the core of lipid metabolism within the skin. An imbalance in lipid metabolism can result in the reduced production of ceramides and other essential lipids, compromising the integrity of the skin’s lipid barrier [54]. This disruption can result in skin dryness, an increased susceptibility to dehydration, and heightened inflammation, visibly affecting the skin’s health and appearance.

### 4.3. Melanocytes

Melanocytes, residing in the basal layer of the epidermis, are the custodians of skin pigmentation, chiefly through the intricate process of melanin biosynthesis [55]. Melanin imparts color to the skin and is pivotal in shielding the skin against the detrimental effects of ultraviolet (UV) radiation [56]. The metabolism of melanocytes plays a pivotal role in skin aging, as it encompasses a complex interplay of glucose, lipid, and protein metabolism. This metabolic orchestra is instrumental in regulating not only the production of melanin but also the overall health of the skin.

The regulation of glucose metabolism in melanocytes is of utmost importance. Glucose serves as the primary energy source for melanin production [57]. Within melanocytes, the tyrosinase enzyme catalyzes the conversion of tyrosine into melanin precursors. This enzymatic process is highly sensitive to glucose levels, and any imbalances can lead to irregularities in melanin production, contributing to variations in skin pigmentation [58].

Furthermore, lipid metabolism within melanocytes is vital for the storage and transport of melanin. Lipids serve as carriers for melanin granules, ensuring their distribution to keratinocytes throughout the epidermis [54]. An imbalance in the lipid metabolism can disrupt this crucial process, leading to irregular melanin dispersion and, subsequently, skin pigmentation issues.

In addition, protein metabolism is essential for the proper functioning of melanocytes. Proteins are involved in melanin synthesis and in protecting melanocytes from oxidative stress induced by UV radiation [59]. Any disruptions in protein metabolism can compromise the melanocytes’ ability to withstand environmental stressors, potentially accelerating the formation of age spots and other pigmentary irregularities.

The metabolism of melanocytes plays a pivotal role in skin aging, as it exerts considerable influence over the uniformity of skin tone and the genesis of dermatological pigmentations. Melanocytes orchestrate a finely tuned metabolic orchestra to sustain an even skin tone [51]. Nevertheless, the relentless march of time and environmental stressors can introduce perturbations into this symphony [60]. Metabolic imbalances can emerge, causing an erratic distribution of melanin. This phenomenon often leads to skin pigmentation irregularities and the unwelcome emergence of skin blemishes, such as age spots and melasma [14].

## 5. Membrane or Cytoskeleton Proteins in Skin Metabolism and Aging

Different cells have specific metabolisms and have different influences on skin aging. However, the detailed mechanisms remain unsolved. Recently, there have been more and more studies on cells indicating that some key membrane or cytoskeleton proteins are extremely important in skin metabolism, tissue integrity, and aging. So, in this section, we review the metabolism and aging characters in several essential membrane or cytoskeleton proteins, including connexins, desmins, and occludins, which are vital in cell-to-cell or intercellular communication, metabolic activities, and aging procession among skin cells.

### 5.1. Connexins and Intercellular Communication

Connexins, a class of channel proteins situated on the cell membrane, play pivotal roles in coordinating and balancing three fundamental metabolic processes: glucose metabolism, protein metabolism, and lipid metabolism.

In glucose metabolism, connexins are crucial for absorbing, transporting, and utilizing intracellular glucose to meet the cell’s energy requirements [61]. By forming intercellular channel connections, connexins enable cells to share metabolic products, including glucose and other sugar molecules. This communication aids in coordinating glucose metabolism and ensures that cells can adapt to changes in energy demands [62]. The dynamic nature of gap junctions allows for cells to rapidly adjust the number of gap junction channels at the plasma membrane in response to external or internal signals. Emerging evidence suggests that ubiquitination plays a critical role in regulating connexin turnover and endocytic processes, affecting the functional statuses of gap junctions.

Connexins are also crucial in facilitating the transfer and exchange of proteins and their metabolic byproducts. This communication is essential for ensuring the coordination of protein synthesis and degradation [63]. Connexin channels between cells enable the sharing of amino acids, peptide chains, and other protein metabolic products, fulfilling the requirements for growth, repair, and regulation. Abnormal connexin function can lead to disruptions in protein metabolism and may be associated with the development of diseases in certain circumstances.

In lipid metabolism, connexins are involved in synthesizing, breaking down, and transporting lipid molecules, meeting the cell’s energy and structural needs [62]. Connexins enable cells to share lipid molecules, including lipoproteins and fatty acids, through intercellular channel connections. This sharing helps to maintain the integrity and functionality of cell membranes and ensures that cells have access to necessary lipid resources. Impaired connexin function can lead to disruptions in lipid metabolism, potentially involving issues like high cholesterol or other lipid-related diseases [61].

As cells age, changes occur in the metabolism of connexins. The expression levels of connexins may decrease, resulting in fewer gap junction channels and reduced metabolic product exchange between cells [64]. Additionally, connexin quality and function may be negatively affected by oxidative stress and other aging-related factors. Inflammation and oxidative stress can lead to abnormal modifications or the degradation of connexins, further disrupting intercellular metabolic communication [65].

### 5.2. Desmin and Cytoskeletal Integrity

Desmin is a crucial component of the cell’s internal structure, and it is indispensable in maintaining skin cells’ morphology and structural integrity. These roles are critical in understanding the cellular basis of skin aging.

Desmin’s influence on glucose metabolism stems from its role in maintaining the cell structure. A well-organized cell structure is crucial for efficient glucose transport, particularly in insulin-responsive tissues like muscles [66]. Disrupted desmin function can lead to structural disarray, affecting the distribution of glucose transporters like GLUT4 and impairing insulin signaling and glucose transport [67].

In terms of protein metabolism, desmin’s function is closely tied to the maintenance of structural integrity. Healthy protein turnover is vital for repairing and maintaining the cytoskeletal framework [68]. Altered desmin can disrupt this balance, leading to protein synthesis and degradation imbalances [69].

In lipid metabolism, desmin’s role is linked to the structural integrity of skin cells. Impaired desmin can compromise lipid metabolism, impacting the synthesis and transport of lipids that are essential for skin health. This disruption can affect lipid synthesis and storage within skin cells, potentially leading to skin-related issues, including impaired skin barrier function [70].

As cells age, the functionality of desmin undergoes changes. This decline in desmin function can disrupt the cell structure, impacting the distribution of glucose transporters, reducing glucose absorption efficiency, and increasing the risk of age-related diabetes [71]. Furthermore, in aging cells, decreased desmin functionality can result in imbalances between protein degradation and synthesis, leading to the accumulation of abnormal proteins that are commonly observed in age-related diseases [72]. The diminishing functionality of desmin in aging cells can disrupt the cell structure, further affecting the lipid metabolism in skin cells [73]. This disruption can result in skin-related issues, including impaired skin barrier function. Desmin’s central role in maintaining cell metabolism and structure remains critical, especially in addressing age-related metabolic concerns.

### 5.3. Occludins and Barrier Function

Occludins, as crucial players in forming intercellular tight junctions, are integral to maintaining the skin’s barrier function as a protective barrier against water loss and the intrusion of external agents [74]. This function becomes particularly significant in skin aging, making the skin more susceptible to environmental stressors like UV radiation, pollution, and pathogens. These stressors accelerate aging by compromising collagen integrity and inducing oxidative stress. The skin’s resilience against these aggressors heavily relies on the integrity of occludins and the tight junctions that they support. Disrupted tight junctions can increase skin permeability, rendering it more vulnerable to damage [75].

In the realm of metabolism, the connection between occludins and sugar, protein, and lipid metabolism is evident. Elevated blood glucose levels can adversely affect occludins’ integrity and tight junctions. High glucose levels can trigger the formation of advanced glycation end products (AGEs), potentially interfering with occludin function [76]. Metabolic disruptions in sugar metabolism can also lead to oxidative stress, causing damage to lipids within the skin’s barrier and compromising its quality. Therefore, stable sugar metabolism is essential for occludins to fulfill their roles in maintaining the skin’s barrier function [77].

Imbalances in protein metabolism can directly affect skin proteins, including occludins. With age, accelerated protein degradation, potentially involving occludins, can disrupt tight junctions and make the skin more vulnerable to external aggressors [78]. Maintaining a balance between protein synthesis and degradation is critical to preserve occludin function and the integrity of the skin’s barrier [79]. In lipid metabolism, imbalances can alter the skin’s lipid composition, which is crucial for the skin barrier. Changes in the lipid composition can interfere with the function of tight junction proteins like occludins, ultimately affecting the skin barrier’s integrity. Maintaining the health of the lipid layer is essential to ensure that occludins can uphold tight junctions and preserve the skin’s barrier function [80]. Recent research by Yuan et al. has shed light on the potential benefits of tranexamic acid (TA) in supporting occludins and the skin barrier. Their study demonstrated that TA can significantly expedite skin barrier recovery and boost occludin levels in response to physicochemical damage [81]. This suggests that interventions aimed at enhancing occludin levels, such as using TA, may hold promise in preserving the skin’s barrier function and countering the effects of skin aging.

Furthermore, it is worth noting that occludins, as guardians of skin barrier function, may extend beyond their role in preserving skin integrity. Recent research suggests that occludins may be part of a complex network involved in metabolic regulation, impacting intercellular connections [82]. While the direct influence of occludins on metabolism remains a subject of debate, there are indications that they may play significant roles in intercellular signaling and metabolic pathways [83].

Some studies have hinted at the interaction between occludins and intracellular signaling pathways that are closely related to metabolic regulation. For instance, occludins might indirectly regulate intracellular metabolic responses by influencing the transmission of intercellular signaling molecules [84]. Although these findings require further in-depth research for confirmation, they provide initial clues to the potential link between occludins and metabolism.

In conclusion, occludins, which are essential for skin barrier function, not only prevent water loss and external agent intrusion but may also play vital roles in intercellular signaling and metabolic regulation. Ongoing research in this field is continually evolving, necessitating further experiments and investigations to gain a comprehensive understanding of the complex relationship between occludins and metabolism.

## 6. Anti-Aging Strategies Based on Metabolic Regulation

Based on the above relationship between skin aging metabolism and aging, we next reviewed the literature on anti-skin-aging drugs or active components based on metabolic regulation from existing published research papers and summarized them as follows:

### 6.1. Inhibiting Skin Glycation

AGEs play an important role in skin glycation damage. Reducing the formation of AGEs can resist skin aging [85]. Aminoguanidine (AG) prevents protein modification through advanced Maillard reactions and is a glycosylation inhibitor. A study selected 344 rats for drug treatment (1 g/L), recorded the effects of taking the drug from 6 months to 24 months, and evaluated age-related collagen and glycation items. The results indicate that AG can reduce the loss of collagen, indicating that lowering blood glucose can reduce the degree of skin aging [86]. This comprehensive investigation assessed age-related collagen levels and glycation parameters. In essence, reducing AGE formation emerges as a promising strategy for skin aging prevention, with compounds like aminoguanidine (AG) showing considerable potential as glycosylation inhibitors.

### 6.2. Increasing Skin Protein Levels

Skin proteins, e.g., collagen and membrane or other cytoskeleton proteins (such as connexins, desmins, and occludins), play important roles in skin structure and aging. However, most tactics are focused on how to increase collagen levels. Below are some tactics on how to increase skin collagen.

#### 6.2.1. Inhibiting Collagen Degradation

Skin aging is intricately linked to collagen degradation, resulting in diminished skin elasticity and the formation of unsightly wrinkles. These processes are often orchestrated by the upregulation of matrix metalloproteinases (MMPs). Consequently, a variety of approaches have been developed to counteract collagen degradation, primarily through the inhibition of MMP activity.

For instance, a study involving 22 Asian women’s forearms, treated with hydrolyzed collagen tripeptide CTP over a 4-week period, demonstrated a notable reduction in MMP expression and a corresponding increase in collagen levels, effectively retarding cellular aging [40].

Resveratrol, a natural compound, exhibits the capability to reduce MMP expression and thwart collagen degradation by inhibiting pathways mediated by reactive oxygen species (ROS), such as MAPK and COX-2. This action provides photoprotective effects against skin aging induced by UVB radiation [87].

Eighteen β- Glycyrrhetinic acid derivatives can resist skin aging caused by UVB in doses of 10 and 25 μ M, possess good antioxidant activities [88], resist apoptosis, prevent collagen degradation, and significantly restore UVB-induced damage [89]

AYAPE (Ala-Tyr-Ala-Pro-Glu) is a pentapeptide isolated from Isochrysis zhanjiangensis. The biological activity of AYAPE against skin aging was assessed using UVB-induced HaCat cells. Studies on UVB-induced HaCat cells have found that AYAPE has the potential to inhibit the production of MMP-1, indicating its role in inhibiting collagen degradation in skin cells, a compound that effectively slows skin aging [90].

In human foreskin fibroblasts (HFF-1), Dendrobium nobile Linel polysaccharide (DNLP) was used to measure ROS, MDA, cell viability and lifespan, SOD, CAT, and GSH-Px. DNLP reduces the rate of β-galactosidase staining, mitigates the activation of the JNK/c-Fos/c-Jun pathway, and effectively inhibits MMP expression [91].

The administration of estrogen can significantly delay skin aging. By inhibiting both the expression of MMPs and the degradation of collagen, estrogen plays a role in delaying skin aging [92]. These interventions collectively contribute to our understanding of the strategies available for addressing skin aging and retaining skin vitality.

#### 6.2.2. Promoting Collagen Synthesis

Skin aging is a multifaceted process that not only involves the degradation of collagen but also encompasses a decline in collagen synthesis. To address this aspect of aging, various strategies have been explored, with a focus on enhancing collagen synthesis to maintain skin health [22].

Ascorbic acid (AA), an essential nutrient, is known for its remarkable ability to reduce oxidative stress and stimulate collagen expression. AA plays a significant role in mitigating the effects of skin aging by promoting collagen synthesis [93].

Chondroitin phosphate (CS) has been identified as an agent that not only boosts the proliferation of keratinocytes and fibroblasts but also triggers the migration and synthesis of extracellular matrix (ECM) components in fibroblasts. Its influence on collagen synthesis is channeled through the activation of the extracellular signal-regulated kinase pathway, leading to the expression of type I procollagen. This counteracts collagen loss and effectively resists skin aging [94].

The assessment of extracellular matrix components such as collagen, elastin, and glycosaminoglycan has proven to be instrumental in understanding the dynamics of skin aging. Components like L-fucose and chondroitin sulfate have demonstrated their capacity to protect the skin. They provide essential building blocks for glycosaminoglycans (GAGs), thereby enhancing the production of collagen and elastin in the extracellular matrix.

Human adipose-derived stem cells (ADSCs) are used to treat skin aging by isolating exosomes from ADSC culture medium. ADSC-derived exosomes (ADSC Exos) can alleviate the aging of human dermal fibroblasts (HDF), and ADSC Exos can increase the expression level of type I collagen and reduce reactive oxygen species (ROS) and the positive rate of β- Galactosidase (SA-β-Gal). Moreover, they downregulate the expression of aging-related proteins, including p53, p21, and p16. This demonstrates the ability of extracellular vesicles to modulate skin cell metabolism and counteract the aging process [95].

Collagen peptides derived from poultry and chicken bones, often containing molecules with a molecular weight below 3000 Da, have shown profound effects on aging skin. These peptides contribute to the reduction in skin oxidation levels, the inhibition of AP-1 expression, and the activation of the TGF-β/Smad signaling pathway, all of which culminate in enhanced collagen synthesis. The experimental results attest to the anti-aging potential of these collagen peptides [96].

Blood donation has the remarkable impact of augmenting skin thickness and elevating collagen content, thereby significantly modifying the pathways associated with skin aging in mouse models. This results in the reduction in inflammatory factors such as Cox-2 and IL-1β. Moreover, it leads to an upregulation in the expression of genes related to collagen production. Therefore, judicious blood donation practices can effectively stimulate collagen synthesis and ameliorate skin aging [97].

Aging is associated with progressive skin fragility and tearing tendency, and transcription factor NRF2 is a key regulatory factor for cellular antioxidant response. The genetic activation of NRF2 in mouse fibroblasts inhibits the expression of collagen and elastin, leading to direct overexpression in mouse skin [98]. Pectin can also protect BALB/c-nu mice from UVB-induced skin aging through the NRF2 pathway [99].

Oral hydrolyzed collagen has a significant effect on improving cellular behavior by enhancing protein folding and DNA repair. Collagen beverages can inhibit ROS [100], promote extracellular matrix protein synthesis, and enhance mitochondrial activity. The supplementation of collagen can reduce oxidative damage, improve cell function, and resist skin aging [101].

#### 6.2.3. Simultaneously Inhibiting Collagen Degradation and Promoting Collagen Synthesis

The intricate balance between collagen degradation and synthesis significantly influences the skin’s collagen levels, and certain tactics aim to simultaneously inhibit degradation and promote synthesis. This dual approach offers valuable insights into combating skin aging with a focus on collagen maintenance.

Tendon enzyme C (TNC), a constituent of the extracellular matrix (ECM), holds a pivotal role in various tissues, including the skin. TNC, particularly its TNC-L and TNC-S variants, orchestrates an upregulation of type I collagen expression while concurrently downregulating MMP-1 expression in fibroblasts. Furthermore, TNC induces an increase in TGF-β mRNA levels, which, in turn, activates the TGF-β signaling pathway. This cascade of events culminates in an elevated expression of type I collagen, contributing to ECM integrity and the prevention of skin aging [102].

The production of proteins within the ECM stands as a fundamental component in preserving normal skin structure and delaying skin aging. Copper, which is recognized for its role as a catalyst, is known to stimulate the synthesis of ECM proteins. Notably, Cu^2+^ exhibits a dose-dependent increase in the gene expressions of collagen and elastin. This phenomenon signifies the potential of a combination of amino acids and Cu^2+^ in fostering the synthesis of ECM proteins within dermal fibroblasts. While copper’s role in collagen degradation is not explicitly mentioned, it plays a significant role in promoting collagen synthesis.

Collagen and elastin are prominent constituents of skin evaluation, with both playing crucial roles in maintaining skin health. Collagen peptides and elastin peptides have garnered widespread use for their anti-inflammatory effects. Employing a combination of these two compounds can effectively address skin aging induced by factors like D-galactose and ultraviolet radiation. The mechanism underlying this therapeutic effect involves the upregulation of hyaluronic acid and hydroxyproline expression, along with the inhibition of MMPs and IL-1α. This combined approach enhances skin health by supporting the extracellular matrix and mitigating skin aging [103].

In summary, strategies to increase skin collagen levels are vital for combating the signs of skin aging. Collagen is a critical structural protein contributing to skin elasticity and a youthful appearance. There are various approaches to achieve this goal, such as inhibiting collagen degradation, promoting collagen synthesis, or employing both strategies. Numerous studies have supported these methods and have shown promising results in reducing the visible effects of skin aging. Recently, it was found that membrane or cytoskeleton proteins such as connexins, desmins, and occludins not only serve as structural proteins in the skin but also affect metabolism and aging. However, related products are expected in the future.

### 6.3. Regulating Skin Lipid Metabolism

Lipid metabolism is associated with skin aging. On the one hand, skin lipids play important roles in supporting the skin structure and protecting the barrier. On the other hand, lipid metabolism disorders themselves can affect cell function, leading to skin aging. The beneficial role of subcutaneous adipose tissue in skin rejuvenation stems from its ability to fill the lower volume. Hyaluronic acid (HA) is the main component of the extracellular matrix, and experimental data have shown that cross-linked HA can promote cell adhesion and retain the ability of preadipocytes to generate fat in long-term cell culture. This provides a strategy for preventing facial volume decline during natural skin aging [104].

The regulation of lipid metabolism disorders in the skin is also an important strategy. A study examining the effects of facial and neck skin lipids on women aged 50–69 years showed that retinol can reduce surface lipids and increase skin regeneration ability. This demonstrates that retinol possesses the ability to alleviate the symptoms of skin aging [105]. By improving the lipid metabolism, the vitality of subcutaneous adipocytes is boosted, demonstrating that lipid metabolism regulation can impact skin aging [106]. Improving the gene transcription of cholesterol and fatty acid synthase is important for the integrity of the skin permeability barrier and can improve skin aging [107].

In conclusion, regulating the skin’s lipid metabolism represents a crucial strategy in the fight against skin aging. Skin lipids play a dual role: supporting the skin’s structural integrity and acting as protective barriers. Disorders in lipid metabolism can impair cell function and contribute to aging. Strategies focused on restoring lipid levels and optimizing lipid metabolism have demonstrated potential in alleviating the effects of skin aging. Additionally, addressing lipid metabolism is essential for maintaining the vitality of subcutaneous adipocytes and preventing facial volume loss during the natural aging process. Ultimately, improving gene transcription related to cholesterol and fatty acid synthesis contributes to the integrity of the skin’s permeability barrier and counteracts the signs of skin aging.

### 6.4. Regulating Mitochondrial Energy Metabolism in the Skin

Aging skin cells can undergo changes in the metabolism of substances such as glucose, proteins, and lipids. On one hand, alterations in the metabolism of these substances, such as reduced efficiency, can promote the onset of aging. Conversely, the accelerated metabolism of these compounds can also significantly impact skin aging [108]. The excessive production of reactive oxygen species (ROS) stemming from the heightened metabolism of glucose, proteins, and lipids via mitochondrial energy pathways can inflict damage on body tissues [109]. Therefore, implementing appropriate calorie restriction can inhibit the generation of mitochondrial ROS, subsequently decelerating the aging process and mitigating age-related diseases, ultimately benefiting skin health [110].

Metformin is a widely used hypoglycemic drug with global recognition [111]. Metformin can reduce inflammation by increasing autophagy and improving mitochondrial function [112] and can inhibit skin aging by reducing oxidative stress and mitochondrial dysfunction [113].

Supplementing nicotinamide can restore cellular NAD+ pool and mitochondrial energy, regulate mitochondrial metabolism, and weaken oxidative stress and inflammatory response, and it has the effect of improving skin aging [114,115].

Vitamin D can alleviate oxidative stress and delay skin aging, mainly by inducing the expressions of Nrf2 and Klotho to improve mitochondrial homeostasis. It is a drug that delays skin aging [116].

The regulation of mitochondrial energy metabolism within the skin emerges as a pivotal strategy in the battle against skin aging. The metabolic shifts related to substances such as glucose, proteins, and lipids, whether there is reduced efficiency or an accelerated metabolism, significantly impact skin aging. The excessive generation of ROS through mitochondrial energy metabolism can lead to oxidative stress, a prominent factor in skin aging. Therefore, interventions promoting mitochondrial health and energy regulation promise to mitigate skin aging effects. Compounds like metformin, nicotinamide, and vitamin D exhibit the potential to enhance mitochondrial function, reducing oxidative stress and delaying skin aging. Proper calorie restriction can also inhibit mitochondrial ROS production, contributing to skin rejuvenation. Understanding the profound connection between mitochondrial energy metabolism and skin aging may generate novel strategies and interventions, offering hope for more effective anti-aging solutions.

## 7. Discussions and Conclusions

This paper mainly explores the relationship and related characteristics between skin aging and metabolism from the perspectives of glucose metabolism, protein (amino acid) metabolism, and lipid metabolism, introduces metabolism and aging in different skin cells and key membrane or cytoskeleton proteins, and reviews drugs based on the regulation of the metabolic disorders mentioned above. We found that the metabolic disorders of glucose, proteins, and lipids were closely correlated with aging in the skin.

Different skin cells such as keratinocytes, fibroblasts, and melanocytes exhibit different metabolic characters, usually related to their cell structures and functions (e.g., rapid proliferation and secretion of specific bioactive substances). Besides these, some key membrane proteins like connexins, desmins, and occludins are emerging as proteins that affect metabolism and regulate aging in the skin. So, skin metabolism is very complicated but delicate in the regulation of skin aging.

When considering glucose metabolism, the predominant manifestations encompass a decline in the efficiency of glucose metabolism, an increase in skin glycosylation, and an elevation in the breakdown of fats and proteins. These processes collectively culminate in alterations to the skin structure and function, ultimately giving rise to the emergence of distinctive characteristics associated with skin aging. In terms of protein, the degradation and synthesis of collagen increase, leading to the loss of skin collagen and the appearance of aging characteristics such as wrinkles. The metabolic disorder of amino acids such as glutamine can affect the growth of skin cells and exert protective effects. Regarding lipid metabolism, changes in lipid degradation or synthesis can influence the structure and function of the skin. This, in turn, impacts skin hydration and luster, ultimately affecting the skin aging process. At the same time, other lipid metabolites can also affect the structure and function of the skin, leading to aging.

Glucose metabolism, protein (amino acid) metabolism, and lipid metabolism not only affect cellular material metabolism but also affect the mitochondrial energy metabolism of cells, and they can cross and influence each other. In terms of mitochondrial energy metabolism, excessive energy intake can lead to an increase in mitochondrial metabolic burden and also affect the production of skin ROS, leading to skin aging. Based on the descriptions above, lower glucose levels or protein or lipid metabolism are also associated with the aging process in the skin. Skin tissue metabolism is very complicated. The question of how to accurately regulate skin metabolism to restore it to normal levels is a challenge that needs to be faced in the future, or it can be a prerequisite for the application of drugs and products in the future.

Based on the aforementioned metabolic disorders in the skin, we found that many research strategies are mostly focused on improving the skin collagen levels, anti-skin glycation, and regulating skin mitochondrial metabolism. Among them are many studies focused on regulating skin structural support proteins, such as inhibiting collagen degradation and promoting collagen synthesis, and the products involve many biochemical factors. However, few products regulate skin glycosylation, skin lipids, and mitochondrial metabolism. Membrane or skeleton proteins also play important roles in skin metabolism and aging. However, few products were found in the market. We look forward to further breakthroughs in this area in the future.

The skin is composed of the epidermis, basal cell layer, and dermis. Each layer consists of different types of cells and exhibits significant heterogeneity. For example, epidermal cells are composed of keratinocytes, melanocytes, Langerhans cells, and Merkel cells. The dermis is composed of fibroblasts, mast cells, and macrophages. Particularly, although the epidermis only accounts for 5% of the skin, these types of cells have high metabolic activities, usually related to their rapid growths and secretion of specific substances, which might be able to be affected by some drugs or components with metabolic regulation activities. Due to the complexity of skin cells, the specific metabolic characteristics and differences among different skin cells are not yet clear, and further research is needed.

**Figure 1:**
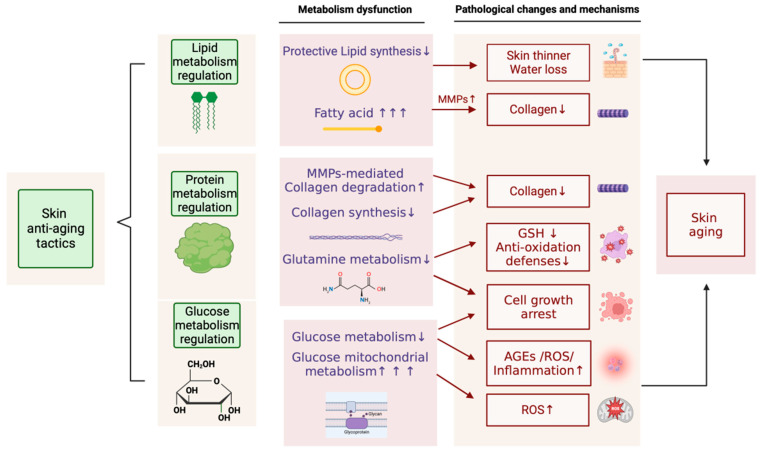
Effects of metabolism on skin aging and potential anti-aging tactics.

In conclusion, skin aging and metabolism are intricately linked and deeply interdependent. The main ideas are summarized in Figure 1. Regulating specific metabolic disorders in the skin has become a promising approach for anti-aging strategies. At the same time, most strategies and products currently use collagen as the core for skin structural support. More interventions must be targeted at skin glycosylation inhibition, amino acid metabolism regulation, lipid metabolism regulation, and even membrane or other cytoskeleton proteins. However, the precise regulation of skin cell metabolism is still a huge challenge. Specific molecular targets for production development are relatively lacking. Future researchers should pay more attention to basic research and production development based on different skin cell metabolisms and molecular targets in tackling the topic of skin aging.
